# A one-dimensional polymeric cobalt(III)–potassium complex with 18-crown-6, cyanide and porphyrinate ligands

**DOI:** 10.1107/S1600536814003596

**Published:** 2014-02-26

**Authors:** Yassine Belghith, Hamza Toumi, Jean-Claude Daran, Habib Nasri

**Affiliations:** a Laboratoire de Physico-chimie des Matériaux, Faculté des Sciences de Monastir, Avenue de l’environnement, 5019 Monastir, University of Monastir, Tunisia; b Laboratoire de Chimie de Coordination CNRS UPR 8241, 205 Route de Narbonne, 31077 Toulouse Cedex 04, France

## Abstract

The reaction of Co^II^(TpivPP) {TpivPP is the dianion of 5,10,15,20-tetra­kis­[2-(2,2-di­methyl­propanamido)­phen­yl]por­ph­yrin} with an excess of KCN salts and an excess of the 18-crown-6 in chloro­benzene leads to the polymeric title compound *catena*-poly[[dicyanido-2κ^2^ *C*-(1,4,7,10,13,16-hexa­oxa­cyclo­octa­decane-1κ^6^ *O*){μ_3_-(2α,2β)-5,10,15,20-tetra­kis­[2-(2,2-di­methyl­propanamido)­phen­yl]porphyrinato-1κ*O* ^5^:2κ^4^ *N*,*N*′,*N*′′,*N*′′′:1′κ*O* ^15^}cobalt(III)potassium] dihydrate], {[CoK(CN)_2_(C_12_H_24_O_6_)(C_64_H_64_N_8_O_4_]·2H_2_O}_*n*_. The Co^III^ ion lies on an inversion center, and the asymmetric unit contains one half of a [Co^III^(2α,2β-TpivPP)(CN)_2_]^−^ ion complex and one half of a [K(18-C-6]^+^ counter-ion (18-C-6 is 1,4,7,10,13,16-hexa­oxa­cyclo­octa­deca­ne), where the K^I^ ion lies on an inversion center. The Co^III^ ion is hexa­coordinated by two C-bonded axial cyanide ligands and the four pyrrole N atoms of the porphyrin ligand. The K^I^ ion is chelated by the six O atoms of the 18-crown-6 mol­ecule and is further coordinated by two O atoms of pivalamido groups of the porphyrin ligands, leading to the formation of polymeric chains running along [011]. In the crystal, the polymeric chains and the lattice water mol­ecules are linked by N—H⋯O and O—H⋯N hydrogen bonds, as well as weak C—H⋯O, O—H⋯π and C—H⋯π inter­actions into a three-dimensional supra­molecular architecture.

## Related literature   

For the synthesis, see: Collman *et al.* (1978 ▶). For related structures, see: Iimuna *et al.* (1988 ▶); Hoshino *et al.* (2000 ▶); Konarev *et al.* (2003 ▶); Ali *et al.* (2011 ▶); Pratt (1972 ▶); Li *et al.* (2010 ▶). For a description of the Cambridge Structural Database, see: Allen (2002 ▶).
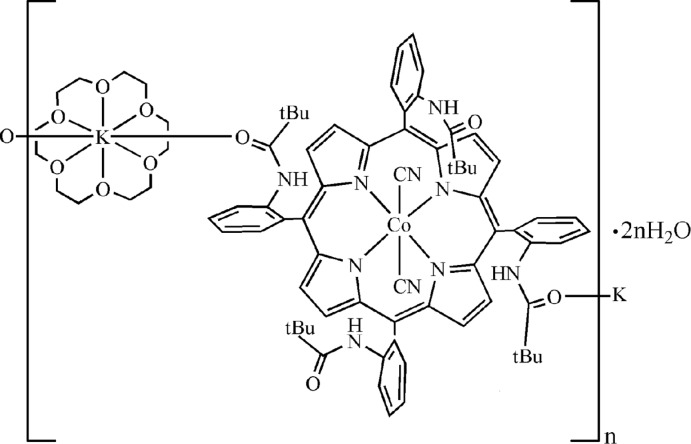


## Experimental   

### 

#### Crystal data  

[CoK(CN)_2_(C_12_H_24_O_6_)(C_64_H_64_N_8_O_4_]·2H_2_O
*M* *_r_* = 1459.70
Triclinic, 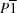

*a* = 9.1885 (3) Å
*b* = 14.4631 (4) Å
*c* = 14.6845 (4) Å
α = 98.342 (2)°
β = 102.170 (2)°
γ = 93.101 (2)°
*V* = 1880.20 (10) Å^3^
*Z* = 1
Mo *K*α radiation
μ = 0.35 mm^−1^
*T* = 180 K
0.48 × 0.40 × 0.30 mm

#### Data collection  

Agilent Xcalibur (Eos, Gemini ultra) diffractometer
Absorption correction: multi-scan (*CrysAlis PRO*; Agilent, 2012 ▶) *T*_min_ = 0.86, *T*_max_ = 0.90
38025 measured reflections
7400 independent reflections
5986 reflections with *I* > 2σ(*I*)
*R*_int_ = 0.034

#### Refinement  

*R*[*F* ^2^ > 2σ(*F* ^2^)] = 0.036
*wR*(*F* ^2^) = 0.105
*S* = 1.07
7400 reflections
475 parameters
2 restraints
H atoms treated by a mixture of independent and constrained refinement
Δρ_max_ = 0.38 e Å^−3^
Δρ_min_ = −0.26 e Å^−3^

### 

Data collection: *CrysAlis PRO* (Agilent, 2012 ▶); cell refinement: *CrysAlis PRO*; data reduction: *CrysAlis PRO*; program(s) used to solve structure: *SIR2004* (Burla *et al.*, 2005 ▶); program(s) used to refine structure: *SHELXL97* (Sheldrick, 2008 ▶); molecular graphics: *ORTEPIII* (Burnett & Johnson, 1996 ▶) and *ORTEP-3 for Windows* (Farrugia, 2012 ▶); software used to prepare material for publication: *WinGX* (Farrugia, 2012 ▶).

## Supplementary Material

Crystal structure: contains datablock(s) I, New_Global_Publ_Block. DOI: 10.1107/S1600536814003596/xu5770sup1.cif

Structure factors: contains datablock(s) I. DOI: 10.1107/S1600536814003596/xu5770Isup2.hkl

CCDC reference: 987431

Additional supporting information:  crystallographic information; 3D view; checkCIF report

## Figures and Tables

**Table 1:** Selected bond lengths (Å)

Co—N1	1.9853 (13)
Co—N2	1.9834 (14)
Co—C33	1.9129 (18)
K—O2^i^	2.7789 (15)
K—O3	2.8633 (13)
K—O4	2.7917 (13)
K—O5	2.7505 (13)

Symmetry code: (i) 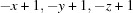.

**Table 2:** Hydrogen-bond geometry (Å, °) *Cg*1, *Cg*2, *Cg*3 and *Cg*5 are the centroids of the N1/C2–C5, N2/C7–C10, Co/N1/C2/C1/C10′/N2′ and Co/N2/C10/C1′/C2′/N1′ rings respectively.

*D*—H⋯*A*	*D*—H	H⋯*A*	*D*⋯*A*	*D*—H⋯*A*
N3—H*N*3⋯O6	0.88	2.09	2.966 (2)	172
O6—H1*O*6⋯N5^ii^	0.87	1.95 (2)	2.810 (3)	172 (2)
C20—H20*C*⋯O6	0.98	2.51	3.413 (3)	153
O6—H2*O*6⋯*Cg*2^iii^	0.88	2.73 (2)	3.272 (2)	121 (2)
O6—H2*O*6⋯*Cg*3	0.88	2.81 (2)	3.455 (2)	131 (2)
O6—H2*O*6⋯*Cg*5^iii^	0.88	2.81 (2)	3.455 (2)	131 (2)
C21—H21*B*⋯*Cg*1^iv^	0.98	2.82	3.737 (3)	156

Symmetry codes: (ii) 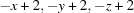; (iii) 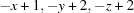; (iv) 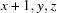.

**Fig. 1.**
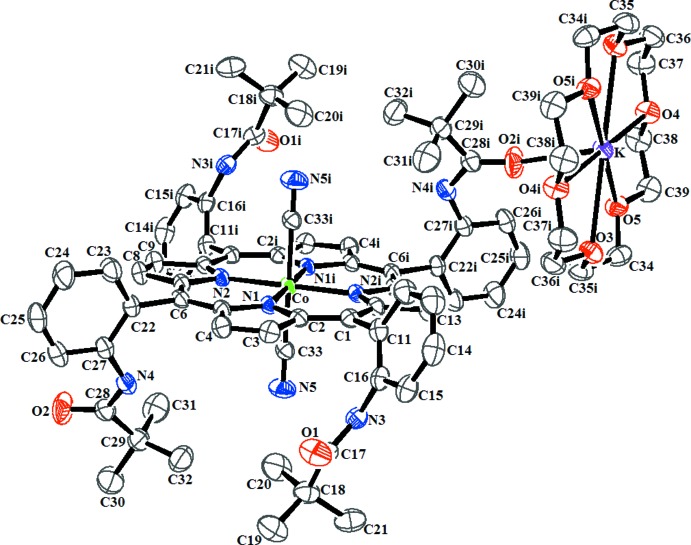
An ORTEP view of the molecular structure of the title molecule with the atom-numbering. Displacement ellipsoids are drawn at 50%. The H atoms have been omitted for clarity.

**Fig. 2.**
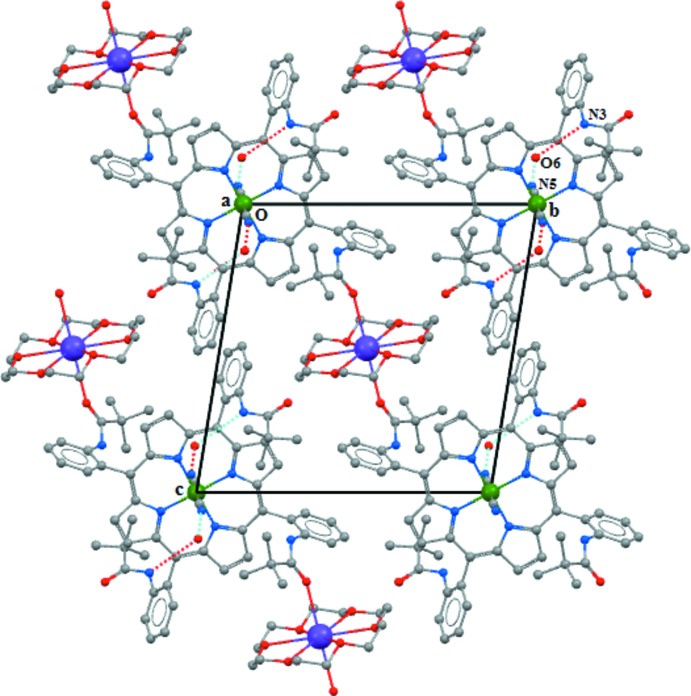
The crystal structure of the title compound plotted in projection along [100]. H atoms have been omitted.

## supplementary crystallographic information

### 1. Comment

In the Cambridge Structural Database (CSD, Version 5.35; Allen, 2002) there are more than ninety structures of cyano-metalloporphyrins. This large number of structures reflects the importance of this type of compounds. Nevertheless, only one structure of cyano-porphyrin species with a cobalt as central ion is known (Hoshino *et al.*., 2000). The cyano-cobalt porphyrin derivatives are good model for the B12 vitamin called cobalamin, which is a cobalt porphyrin-like protein responsible, inter alia, of the formation of blood.

We reports herein the crystal structure of the poly[(1,4,7,10,13,16-hexaoxacyclooctadecane)potassium(+)(dicyano)(2*α*,2*β*-5,10,15,20-tetrakis[2-(2,2-dimethylpropanamido)-phenyl]porphyrinato-κ^4^N,N',N'',N''')cobaltate(III) dihydrate] with formula{[K(18-C-6)][Co^III^(2α,2β-TpivPP)(CN)_2_].2H_2_O}_n_.

In this complex, the cobalt is coordinated to the four N atoms of the porphyrin ring and the carbons of the two trans cyano axial ligands (Fig. 1).

It has been noticed that there is a relationship between the ruffling of the porphyrinato core and the mean equatorial Co—N_p_ distance; the porphyrinato core is ruffled as the Co—N_p_ distance decreases (Iimuna *et al.*, 1988). Indeed, for the very ruffled structure [Co^II^(TPP)] (Konarev *et al.*, 2003) the Co—N_p_ bond length value is 1.923 (4) Å while the practically planar porphyrin core of the ion complex [Co^III^(OEP)(NO_2_)_2_]^-^ (OEP is the dianion of the octaethylporphyrin; Ali *et al.*, 2011) presents a Co—N_p_ distance of 1.988 (2) Å. Therefore, the Co—N_p_ bond length in the title complex [1.9844 (14) Å] is normal for a cobalt planar porphyrin species. It is noteworthy that the related dicyano-cobalt(III) derivative

{[K(18-C-6)H_2_O]_2_[(CN)_2_Co^III^(TPP)]}[(CN)_2_Co^III^(TPP)].C_7_H_8_ (Hoshino *et al.*, 2000) exhibits a very short Co^__^N_p_ distance [1.93 (1) Å] which is in accordance with a very ruffled porphyrin core.

The Co—C(cyano) bond length value [1.9129 (18) Å] is very close to that of vitamin B12 (1.92 Å ) (Pratt, 1972). This distances is slightly shorter compared to those of the related dicyano-cobalt species mentioned above [Co—C(CN) = 1.98 (2) Å and 1.94 (2) Å].

The potassium anion is coordinated to the six oxygen atoms of the 18-crown-6 where the K—O bond length values are in the range [2.7505 (13) Å - 2.8633 (13) Å]. This cation is also linked to the oxygen O2 of one pivalamido group of the 2α,2β-TpivPP porphyrin with a K^__^O2 distance of 2.7789 (15) Å leading to a 1D coordination polymer. One water molecule is linked to the nitrogen atom (N3) of one pivalamido group and the nitrogen N5 of the cyano axial ligand via the two intramolecular hydrogen bonds N3-HN3···O6 [2.966 (3) Å] and O6-H1O6···N5 [2.810 (3) Å].

The crystal packing features weak C—H···π interactions between the 1D polymer chains (Table 1 and Fig. 2).

An interesting phenomenon concerning the structure title compound where the porphyrin starting material is the atropisomer α,α,α,α-TpivPP but the final product contains the 2*α*,2*β*-TpivPP atropisomer in the polymer {[K(18-C-6)][Co^III^(2α,2β-TpivPP)(CN)_2_].2H_2_O}_n_. This kind of stereoisomerism of the TpivPP porphyrin was mentioned in the literature (Li *et al.*, 2010).

### 2. Experimental

To a solution of [Co^II^(TpivPP)] (Collman *et al.*, 1978) (100 mg, 0.067 mmol) in chlorobenzene (10 mL) was added an excess of 18-crown-6 (150 mg, 0.567 mmol) and potassium cyanide (100 mg, 0.378 mmol). A rapid color change from orange-red to green occurred. The resulting material was crystallized by diffusion of hexanes through the chlorobenzene solution which yields {[K(18-C-6)][Co^III^(2α,2β-TpivPP)(CN)_2_].2H_2_O}_n_ crystals as synthesis product.

### 3. Refinement

The two hydrogens of the water molecule were found in the difference Fourier map and were included in the refinement using restraints (O-H = 0.85 (1) Å) with U_iso_(H) = 1.2U_eq_(O6). Other H atoms attached to C and N atoms were fixed geometrically and treated as riding with C—H = 0.99 Å (methylene), 0.95 Å (aromatic) and 0.98 Å with *U*_iso_(H) = 1.2*U*_eq_(C_aromatic, methylene, methyl_) and N—H = 0.88 Å with *U*_iso_(H) = 1.2*U*_eq_(N).

### Crystal data

**Table:**

[CoK(CN)_2_(C_12_H_24_O_6_)(C_64_H_64_N_8_O_4_]·2H_2_O	*Z* = 1
*M**_r_* = 1459.70	*F*(000) = 772
Triclinic, *P*1	*D*_x_ = 1.289 Mg m^−^^3^
Hall symbol: -P 1	Mo *K*α radiation, λ = 0.71073 Å
*a* = 9.1885 (3) Å	Cell parameters from 7400 reflections
*b* = 14.4631 (4) Å	θ = 2.9–26.1°
*c* = 14.6845 (4) Å	µ = 0.35 mm^−^^1^
α = 98.342 (2)°	*T* = 180 K
β = 102.170 (2)°	Prism, dark purple
γ = 93.101 (2)°	0.48 × 0.40 × 0.30 mm
*V* = 1880.20 (10) Å^3^	

### Data collection

**Table:**

Agilent Xcalibur (Eos, Gemini ultra) diffractometer	7400 independent reflections
Radiation source: Enhance (Mo) X-ray Source	5986 reflections with *I* > 2σ(*I*)
Graphite monochromator	*R*_int_ = 0.034
Detector resolution: 16.1978 pixels mm^-1^	θ_max_ = 26.0°, θ_min_ = 3.0°
ω scans	*h* = −11→11
Absorption correction: multi-scan (*CrysAlis PRO*; Agilent, 2012)	*k* = −17→17
*T*_min_ = 0.86, *T*_max_ = 0.90	*l* = −18→18
38025 measured reflections	

### Refinement

**Table:**

Refinement on *F*^2^	Primary atom site location: structure-invariant direct methods
Least-squares matrix: full	Secondary atom site location: difference Fourier map
*R*[*F*^2^ > 2σ(*F*^2^)] = 0.036	Hydrogen site location: inferred from neighbouring sites
*wR*(*F*^2^) = 0.105	H atoms treated by a mixture of independent and constrained refinement
*S* = 1.07	*w* = 1/[σ^2^(*F*_o_^2^) + (0.0598*P*)^2^ + 0.4188*P*] where *P* = (*F*_o_^2^ + 2*F*_c_^2^)/3
7400 reflections	(Δ/σ)_max_ = 0.001
475 parameters	Δρ_max_ = 0.38 e Å^−^^3^
2 restraints	Δρ_min_ = −0.26 e Å^−^^3^

### Special details

**Table:**

Geometry. All s.u.'s (except the s.u. in the dihedral angle between two l.s. planes) are estimated using the full covariance matrix. The cell s.u.'s are taken into account individually in the estimation of s.u.'s in distances, angles and torsion angles; correlations between s.u.'s in cell parameters are only used when they are defined by crystal symmetry. An approximate (isotropic) treatment of cell s.u.'s is used for estimating s.u.'s involving l.s. planes.
Refinement. Refinement of *F*^2^ against ALL reflections. The weighted *R*-factor *wR* and goodness of fit *S* are based on *F*^2^, conventional *R*-factors *R* are based on *F*, with *F* set to zero for negative *F*^2^. The threshold expression of *F*^2^ > σ(*F*^2^) is used only for calculating *R*-factors(gt) *etc*. and is not relevant to the choice of reflections for refinement. *R*-factors based on *F*^2^ are statistically about twice as large as those based on *F*, and *R*- factors based on ALL data will be even larger.

### Fractional atomic coordinates and isotropic or equivalent isotropic displacement parameters (Å^2^)

**Table:**

	*x*	*y*	*z*	*U*_iso_*/*U*_eq_	
Co	0.5000	1.0000	1.0000	0.02133 (10)	
N1	0.52932 (15)	0.88932 (9)	1.06541 (9)	0.0234 (3)	
N2	0.39217 (15)	0.91778 (9)	0.88238 (10)	0.0241 (3)	
N3	0.94155 (17)	0.88392 (11)	1.27847 (11)	0.0329 (3)	
HN3	0.9590	0.9249	1.2424	0.039*	
N4	0.57697 (19)	0.65256 (12)	0.83577 (12)	0.0397 (4)	
HN4	0.6294	0.6952	0.8815	0.048*	
N5	0.7928 (2)	0.97203 (13)	0.93683 (14)	0.0464 (4)	
O1	0.97516 (19)	0.73834 (11)	1.31093 (12)	0.0533 (4)	
O2	0.5711 (2)	0.57652 (13)	0.69039 (11)	0.0646 (5)	
C1	0.66304 (19)	0.96624 (12)	1.22370 (12)	0.0265 (4)	
C2	0.60058 (19)	0.88825 (12)	1.15701 (12)	0.0252 (4)	
C3	0.6008 (2)	0.79372 (13)	1.17555 (13)	0.0316 (4)	
H3	0.6432	0.7742	1.2337	0.038*	
C4	0.5306 (2)	0.73807 (12)	1.09608 (13)	0.0315 (4)	
H4	0.5144	0.6716	1.0869	0.038*	
C5	0.48427 (19)	0.79756 (12)	1.02721 (12)	0.0253 (4)	
C6	0.40387 (19)	0.76506 (12)	0.93650 (12)	0.0262 (4)	
C7	0.36008 (19)	0.82279 (12)	0.87009 (12)	0.0264 (4)	
C8	0.2772 (2)	0.78898 (13)	0.77591 (13)	0.0330 (4)	
H8	0.2400	0.7259	0.7503	0.040*	
C9	0.2623 (2)	0.86297 (13)	0.73112 (13)	0.0331 (4)	
H9	0.2128	0.8623	0.6673	0.040*	
C10	0.33430 (19)	0.94339 (12)	0.79678 (12)	0.0260 (4)	
C11	0.7262 (2)	0.95246 (12)	1.32261 (12)	0.0303 (4)	
C12	0.6505 (2)	0.98329 (15)	1.39281 (14)	0.0403 (5)	
H12	0.5619	1.0140	1.3770	0.048*	
C13	0.7022 (3)	0.96996 (16)	1.48480 (15)	0.0467 (5)	
H13	0.6494	0.9909	1.5319	0.056*	
C14	0.8304 (3)	0.92624 (16)	1.50768 (14)	0.0470 (5)	
H14	0.8657	0.9165	1.5709	0.056*	
C15	0.9088 (2)	0.89617 (14)	1.43992 (14)	0.0394 (5)	
H15	0.9978	0.8661	1.4566	0.047*	
C16	0.8571 (2)	0.91008 (13)	1.34684 (13)	0.0310 (4)	
C17	0.9971 (2)	0.79926 (14)	1.26544 (14)	0.0355 (4)	
C18	1.0893 (2)	0.78598 (16)	1.18982 (14)	0.0421 (5)	
C19	1.1415 (3)	0.68749 (19)	1.18373 (18)	0.0592 (7)	
H19A	1.2030	0.6799	1.2449	0.089*	
H19B	1.2006	0.6780	1.1354	0.089*	
H19C	1.0544	0.6412	1.1670	0.089*	
C20	0.9932 (3)	0.7967 (2)	1.09392 (16)	0.0614 (7)	
H20A	0.9085	0.7486	1.0764	0.092*	
H20B	1.0536	0.7892	1.0461	0.092*	
H20C	0.9560	0.8590	1.0978	0.092*	
C21	1.2242 (3)	0.85800 (19)	1.21753 (18)	0.0542 (6)	
H21A	1.1904	0.9213	1.2210	0.081*	
H21B	1.2852	0.8487	1.1702	0.081*	
H21C	1.2839	0.8504	1.2793	0.081*	
C22	0.3639 (2)	0.66159 (12)	0.90657 (12)	0.0291 (4)	
C23	0.2404 (2)	0.61838 (14)	0.92750 (16)	0.0428 (5)	
H23	0.1823	0.6543	0.9634	0.051*	
C24	0.1995 (3)	0.52378 (15)	0.89726 (17)	0.0509 (6)	
H24	0.1138	0.4950	0.9120	0.061*	
C25	0.2836 (3)	0.47170 (14)	0.84571 (16)	0.0467 (5)	
H25	0.2553	0.4067	0.8243	0.056*	
C26	0.4078 (3)	0.51240 (14)	0.82480 (14)	0.0419 (5)	
H26	0.4662	0.4755	0.7898	0.050*	
C27	0.4487 (2)	0.60767 (13)	0.85471 (13)	0.0319 (4)	
C28	0.6291 (2)	0.63779 (15)	0.75535 (14)	0.0403 (5)	
C29	0.7599 (2)	0.70560 (17)	0.75141 (15)	0.0457 (5)	
C30	0.8447 (3)	0.6566 (2)	0.6829 (2)	0.0762 (9)	
H30A	0.8931	0.6047	0.7094	0.114*	
H30B	0.9209	0.7014	0.6722	0.114*	
H30C	0.7750	0.6321	0.6228	0.114*	
C31	0.6936 (3)	0.79227 (19)	0.71477 (19)	0.0628 (7)	
H31A	0.6252	0.7727	0.6531	0.094*	
H31B	0.7745	0.8362	0.7084	0.094*	
H31C	0.6389	0.8231	0.7595	0.094*	
C32	0.8650 (3)	0.73621 (19)	0.84737 (17)	0.0562 (6)	
H32A	0.8144	0.7761	0.8885	0.084*	
H32B	0.9545	0.7715	0.8397	0.084*	
H32C	0.8938	0.6807	0.8757	0.084*	
C33	0.6838 (2)	0.98363 (12)	0.96064 (12)	0.0290 (4)	
K	0.5000	0.5000	0.5000	0.03158 (14)	
O3	0.22357 (15)	0.57249 (10)	0.52236 (9)	0.0391 (3)	
O4	0.45230 (16)	0.68329 (10)	0.47014 (10)	0.0403 (3)	
O5	0.68531 (15)	0.59387 (9)	0.41262 (10)	0.0381 (3)	
C34	0.8311 (2)	0.56578 (16)	0.41702 (15)	0.0428 (5)	
H34A	0.8960	0.5911	0.4795	0.051*	
H34B	0.8744	0.5901	0.3680	0.051*	
C35	0.1781 (2)	0.53796 (16)	0.59851 (15)	0.0436 (5)	
H35A	0.0793	0.5594	0.6039	0.052*	
H35B	0.2507	0.5630	0.6582	0.052*	
C36	0.2412 (3)	0.67115 (15)	0.53654 (16)	0.0464 (5)	
H36A	0.3102	0.6946	0.5981	0.056*	
H36B	0.1434	0.6963	0.5378	0.056*	
C37	0.3018 (3)	0.70415 (16)	0.45976 (17)	0.0497 (5)	
H37A	0.2425	0.6726	0.3977	0.060*	
H37B	0.2957	0.7726	0.4630	0.060*	
C38	0.5214 (3)	0.71294 (16)	0.40086 (16)	0.0470 (5)	
H38A	0.5153	0.7812	0.4018	0.056*	
H38B	0.4691	0.6800	0.3376	0.056*	
C39	0.6799 (2)	0.69204 (14)	0.42005 (16)	0.0445 (5)	
H39A	0.7307	0.7161	0.3740	0.053*	
H39B	0.7316	0.7230	0.4842	0.053*	
O6	0.97562 (19)	1.03103 (13)	1.16135 (13)	0.0560 (4)	
H1O6	1.053 (2)	1.032 (2)	1.136 (2)	0.084*	
H2O6	0.890 (2)	1.038 (2)	1.1234 (17)	0.084*	

### Atomic displacement parameters (Å^2^)

**Table:**

	*U*^11^	*U*^22^	*U*^33^	*U*^12^	*U*^13^	*U*^23^
Co	0.02503 (18)	0.01677 (16)	0.02392 (17)	0.00123 (12)	0.00987 (13)	0.00257 (12)
N1	0.0261 (7)	0.0199 (7)	0.0256 (7)	0.0015 (5)	0.0096 (6)	0.0030 (6)
N2	0.0294 (8)	0.0185 (7)	0.0268 (7)	0.0021 (6)	0.0119 (6)	0.0031 (6)
N3	0.0344 (9)	0.0331 (8)	0.0345 (8)	0.0009 (7)	0.0114 (7)	0.0117 (7)
N4	0.0441 (10)	0.0353 (9)	0.0378 (9)	−0.0003 (7)	0.0152 (8)	−0.0081 (7)
N5	0.0391 (10)	0.0504 (11)	0.0590 (11)	0.0087 (8)	0.0280 (9)	0.0125 (9)
O1	0.0638 (10)	0.0423 (9)	0.0683 (10)	0.0127 (8)	0.0343 (9)	0.0243 (8)
O2	0.0722 (12)	0.0741 (12)	0.0382 (9)	−0.0237 (9)	0.0190 (8)	−0.0202 (8)
C1	0.0288 (9)	0.0280 (9)	0.0253 (8)	0.0022 (7)	0.0111 (7)	0.0055 (7)
C2	0.0261 (9)	0.0244 (8)	0.0280 (9)	0.0014 (7)	0.0112 (7)	0.0059 (7)
C3	0.0366 (10)	0.0274 (9)	0.0324 (9)	0.0006 (8)	0.0077 (8)	0.0107 (7)
C4	0.0380 (10)	0.0218 (9)	0.0354 (10)	0.0017 (7)	0.0082 (8)	0.0071 (7)
C5	0.0288 (9)	0.0200 (8)	0.0294 (9)	0.0029 (7)	0.0111 (7)	0.0044 (7)
C6	0.0304 (9)	0.0201 (8)	0.0307 (9)	0.0030 (7)	0.0131 (7)	0.0028 (7)
C7	0.0301 (9)	0.0214 (8)	0.0288 (9)	0.0015 (7)	0.0109 (7)	0.0017 (7)
C8	0.0440 (11)	0.0220 (9)	0.0308 (9)	−0.0001 (8)	0.0081 (8)	−0.0018 (7)
C9	0.0422 (11)	0.0287 (9)	0.0271 (9)	0.0019 (8)	0.0073 (8)	0.0010 (7)
C10	0.0294 (9)	0.0242 (9)	0.0261 (9)	0.0021 (7)	0.0115 (7)	0.0018 (7)
C11	0.0380 (10)	0.0261 (9)	0.0274 (9)	−0.0046 (7)	0.0099 (8)	0.0046 (7)
C12	0.0479 (12)	0.0417 (11)	0.0342 (10)	0.0005 (9)	0.0169 (9)	0.0055 (9)
C13	0.0596 (14)	0.0496 (13)	0.0332 (11)	−0.0040 (11)	0.0203 (10)	0.0030 (9)
C14	0.0638 (15)	0.0462 (12)	0.0282 (10)	−0.0153 (11)	0.0082 (10)	0.0069 (9)
C15	0.0446 (12)	0.0362 (11)	0.0346 (10)	−0.0079 (9)	0.0027 (9)	0.0096 (8)
C16	0.0364 (10)	0.0272 (9)	0.0288 (9)	−0.0074 (7)	0.0074 (8)	0.0057 (7)
C17	0.0294 (10)	0.0406 (11)	0.0372 (10)	0.0011 (8)	0.0064 (8)	0.0112 (9)
C18	0.0416 (12)	0.0512 (13)	0.0376 (11)	0.0113 (10)	0.0129 (9)	0.0119 (9)
C19	0.0670 (16)	0.0624 (16)	0.0539 (14)	0.0226 (13)	0.0226 (13)	0.0087 (12)
C20	0.0686 (17)	0.0807 (19)	0.0368 (12)	0.0225 (14)	0.0115 (11)	0.0100 (12)
C21	0.0397 (12)	0.0689 (16)	0.0620 (15)	0.0056 (11)	0.0215 (11)	0.0215 (12)
C22	0.0377 (10)	0.0211 (9)	0.0274 (9)	0.0016 (7)	0.0059 (8)	0.0032 (7)
C23	0.0487 (12)	0.0308 (10)	0.0512 (12)	−0.0017 (9)	0.0213 (10)	0.0006 (9)
C24	0.0595 (14)	0.0321 (11)	0.0621 (15)	−0.0131 (10)	0.0230 (12)	0.0027 (10)
C25	0.0666 (15)	0.0200 (9)	0.0500 (13)	−0.0018 (9)	0.0085 (11)	0.0030 (9)
C26	0.0586 (13)	0.0252 (10)	0.0413 (11)	0.0085 (9)	0.0116 (10)	0.0006 (8)
C27	0.0399 (10)	0.0247 (9)	0.0303 (9)	0.0043 (8)	0.0071 (8)	0.0022 (7)
C28	0.0408 (11)	0.0446 (12)	0.0332 (10)	0.0065 (9)	0.0081 (9)	−0.0021 (9)
C29	0.0418 (12)	0.0561 (14)	0.0371 (11)	−0.0009 (10)	0.0137 (9)	−0.0054 (10)
C30	0.0638 (17)	0.092 (2)	0.0712 (18)	−0.0076 (15)	0.0391 (15)	−0.0223 (16)
C31	0.0665 (17)	0.0651 (17)	0.0558 (15)	−0.0081 (13)	0.0110 (13)	0.0149 (13)
C32	0.0459 (13)	0.0666 (16)	0.0499 (14)	−0.0052 (11)	0.0062 (11)	−0.0014 (12)
C33	0.0339 (10)	0.0238 (9)	0.0307 (9)	0.0018 (7)	0.0105 (8)	0.0046 (7)
K	0.0369 (3)	0.0262 (3)	0.0338 (3)	−0.0001 (2)	0.0138 (2)	0.0039 (2)
O3	0.0444 (8)	0.0407 (8)	0.0360 (7)	0.0079 (6)	0.0158 (6)	0.0073 (6)
O4	0.0450 (8)	0.0373 (8)	0.0433 (8)	0.0077 (6)	0.0140 (6)	0.0143 (6)
O5	0.0345 (7)	0.0349 (7)	0.0473 (8)	−0.0021 (6)	0.0142 (6)	0.0086 (6)
C34	0.0333 (11)	0.0562 (13)	0.0420 (11)	0.0004 (9)	0.0112 (9)	0.0149 (10)
C35	0.0399 (11)	0.0586 (14)	0.0377 (11)	0.0109 (10)	0.0169 (9)	0.0104 (10)
C36	0.0466 (12)	0.0400 (12)	0.0550 (13)	0.0098 (10)	0.0181 (10)	0.0029 (10)
C37	0.0508 (13)	0.0432 (12)	0.0599 (14)	0.0148 (10)	0.0138 (11)	0.0182 (11)
C38	0.0568 (14)	0.0400 (12)	0.0509 (13)	0.0039 (10)	0.0171 (11)	0.0220 (10)
C39	0.0504 (13)	0.0358 (11)	0.0506 (13)	−0.0068 (9)	0.0144 (10)	0.0170 (9)
O6	0.0493 (10)	0.0643 (11)	0.0697 (12)	0.0116 (8)	0.0359 (9)	0.0250 (9)

### Geometric parameters (Å, º)

**Table:**

Co—N1	1.9853 (13)	C21—H21A	0.9800
Co—N1^i^	1.9853 (13)	C21—H21B	0.9800
Co—N2	1.9834 (14)	C21—H21C	0.9800
Co—N2^i^	1.9834 (14)	C22—C23	1.378 (3)
Co—C33	1.9129 (18)	C22—C27	1.392 (2)
Co—C33^i^	1.9129 (18)	C23—C24	1.381 (3)
N1—C5	1.369 (2)	C23—H23	0.9500
N1—C2	1.369 (2)	C24—C25	1.372 (3)
N2—C7	1.367 (2)	C24—H24	0.9500
N2—C10	1.369 (2)	C25—C26	1.368 (3)
N3—C17	1.355 (2)	C25—H25	0.9500
N3—C16	1.416 (2)	C26—C27	1.390 (3)
N3—HN3	0.8800	C26—H26	0.9500
N4—C28	1.359 (2)	C28—C29	1.526 (3)
N4—C27	1.413 (3)	C29—C30	1.519 (3)
N4—HN4	0.8800	C29—C32	1.521 (3)
N5—C33	1.141 (2)	C29—C31	1.540 (4)
O1—C17	1.213 (2)	C30—H30A	0.9800
O2—C28	1.212 (3)	C30—H30B	0.9800
O2—K	2.7789 (15)	C30—H30C	0.9800
C1—C10^i^	1.383 (2)	C31—H31A	0.9800
C1—C2	1.390 (2)	C31—H31B	0.9800
C1—C11	1.492 (2)	C31—H31C	0.9800
C2—C3	1.432 (2)	C32—H32A	0.9800
C3—C4	1.334 (3)	C32—H32B	0.9800
C3—H3	0.9500	C32—H32C	0.9800
C4—C5	1.433 (2)	K—O2^ii^	2.7789 (15)
C4—H4	0.9500	K—O3^ii^	2.8633 (13)
C5—C6	1.381 (2)	K—O3	2.8633 (13)
C6—C7	1.385 (2)	K—O4^ii^	2.7917 (13)
C6—C22	1.500 (2)	K—O4	2.7917 (13)
C7—C8	1.432 (3)	K—O5^ii^	2.7505 (13)
C8—C9	1.334 (3)	K—O5	2.7505 (13)
C8—H8	0.9500	O3—C36	1.407 (3)
C9—C10	1.429 (3)	O3—C35	1.418 (2)
C9—H9	0.9500	O4—C38	1.411 (2)
C10—C1^i^	1.383 (2)	O4—C37	1.413 (3)
C11—C16	1.383 (3)	O5—C34	1.412 (2)
C11—C12	1.395 (3)	O5—C39	1.412 (2)
C12—C13	1.379 (3)	C34—C35^ii^	1.480 (3)
C12—H12	0.9500	C34—H34A	0.9900
C13—C14	1.369 (3)	C34—H34B	0.9900
C13—H13	0.9500	C35—C34^ii^	1.480 (3)
C14—C15	1.382 (3)	C35—H35A	0.9900
C14—H14	0.9500	C35—H35B	0.9900
C15—C16	1.397 (3)	C36—C37	1.484 (3)
C15—H15	0.9500	C36—H36A	0.9900
C17—C18	1.530 (3)	C36—H36B	0.9900
C18—C21	1.520 (3)	C37—H37A	0.9900
C18—C19	1.524 (3)	C37—H37B	0.9900
C18—C20	1.530 (3)	C38—C39	1.480 (3)
C19—H19A	0.9800	C38—H38A	0.9900
C19—H19B	0.9800	C38—H38B	0.9900
C19—H19C	0.9800	C39—H39A	0.9900
C20—H20A	0.9800	C39—H39B	0.9900
C20—H20B	0.9800	O6—H1O6	0.869 (10)
C20—H20C	0.9800	O6—H2O6	0.881 (10)
			
C33—Co—C33^i^	180.000 (1)	C26—C25—C24	120.64 (19)
C33—Co—N2	89.44 (7)	C26—C25—H25	119.7
C33^i^—Co—N2	90.56 (7)	C24—C25—H25	119.7
C33—Co—N2^i^	90.56 (7)	C25—C26—C27	119.98 (19)
C33^i^—Co—N2^i^	89.44 (7)	C25—C26—H26	120.0
N2—Co—N2^i^	180.000 (1)	C27—C26—H26	120.0
C33—Co—N1	89.73 (6)	C26—C27—C22	120.04 (18)
C33^i^—Co—N1	90.27 (7)	C26—C27—N4	122.02 (17)
N2—Co—N1	90.40 (6)	C22—C27—N4	117.93 (16)
N2^i^—Co—N1	89.60 (6)	O2—C28—N4	121.5 (2)
C33—Co—N1^i^	90.27 (7)	O2—C28—C29	122.94 (19)
C33^i^—Co—N1^i^	89.73 (6)	N4—C28—C29	115.55 (17)
N2—Co—N1^i^	89.60 (6)	C30—C29—C32	109.7 (2)
N2^i^—Co—N1^i^	90.40 (6)	C30—C29—C28	107.74 (19)
N1—Co—N1^i^	180.000 (1)	C32—C29—C28	112.59 (19)
C5—N1—C2	105.66 (14)	C30—C29—C31	110.3 (2)
C5—N1—Co	126.80 (11)	C32—C29—C31	109.3 (2)
C2—N1—Co	127.53 (11)	C28—C29—C31	107.20 (18)
C7—N2—C10	105.43 (14)	C29—C30—H30A	109.5
C7—N2—Co	126.81 (12)	C29—C30—H30B	109.5
C10—N2—Co	127.76 (11)	H30A—C30—H30B	109.5
C17—N3—C16	123.67 (16)	C29—C30—H30C	109.5
C17—N3—HN3	118.2	H30A—C30—H30C	109.5
C16—N3—HN3	118.2	H30B—C30—H30C	109.5
C28—N4—C27	127.75 (17)	C29—C31—H31A	109.5
C28—N4—HN4	116.1	C29—C31—H31B	109.5
C27—N4—HN4	116.1	H31A—C31—H31B	109.5
C28—O2—K	152.04 (16)	C29—C31—H31C	109.5
C10^i^—C1—C2	122.97 (16)	H31A—C31—H31C	109.5
C10^i^—C1—C11	118.31 (15)	H31B—C31—H31C	109.5
C2—C1—C11	118.70 (15)	C29—C32—H32A	109.5
N1—C2—C1	126.06 (15)	C29—C32—H32B	109.5
N1—C2—C3	109.87 (15)	H32A—C32—H32B	109.5
C1—C2—C3	124.07 (16)	C29—C32—H32C	109.5
C4—C3—C2	107.40 (16)	H32A—C32—H32C	109.5
C4—C3—H3	126.3	H32B—C32—H32C	109.5
C2—C3—H3	126.3	N5—C33—Co	178.67 (17)
C3—C4—C5	107.01 (16)	O5^ii^—K—O5	180.0
C3—C4—H4	126.5	O5^ii^—K—O2	72.53 (5)
C5—C4—H4	126.5	O5—K—O2	107.47 (5)
N1—C5—C6	126.14 (15)	O5^ii^—K—O2^ii^	107.47 (5)
N1—C5—C4	110.06 (15)	O5—K—O2^ii^	72.53 (5)
C6—C5—C4	123.80 (16)	O2—K—O2^ii^	180.000 (1)
C5—C6—C7	123.56 (16)	O5^ii^—K—O4^ii^	60.15 (4)
C5—C6—C22	118.87 (15)	O5—K—O4^ii^	119.85 (4)
C7—C6—C22	117.55 (16)	O2—K—O4^ii^	94.94 (5)
N2—C7—C6	126.18 (16)	O2^ii^—K—O4^ii^	85.06 (5)
N2—C7—C8	110.27 (15)	O5^ii^—K—O4	119.85 (4)
C6—C7—C8	123.51 (16)	O5—K—O4	60.15 (4)
C9—C8—C7	106.85 (16)	O2—K—O4	85.06 (5)
C9—C8—H8	126.6	O2^ii^—K—O4	94.94 (5)
C7—C8—H8	126.6	O4^ii^—K—O4	180.0
C8—C9—C10	107.43 (16)	O5^ii^—K—O3^ii^	119.74 (4)
C8—C9—H9	126.3	O5—K—O3^ii^	60.26 (4)
C10—C9—H9	126.3	O2—K—O3^ii^	100.93 (5)
N2—C10—C1^i^	126.01 (16)	O2^ii^—K—O3^ii^	79.07 (5)
N2—C10—C9	110.00 (15)	O4^ii^—K—O3^ii^	61.01 (4)
C1^i^—C10—C9	123.91 (16)	O4—K—O3^ii^	118.99 (4)
C16—C11—C12	119.00 (17)	O5^ii^—K—O3	60.26 (4)
C16—C11—C1	122.11 (16)	O5—K—O3	119.74 (4)
C12—C11—C1	118.89 (17)	O2—K—O3	79.07 (5)
C13—C12—C11	121.1 (2)	O2^ii^—K—O3	100.93 (5)
C13—C12—H12	119.5	O4^ii^—K—O3	118.99 (4)
C11—C12—H12	119.5	O4—K—O3	61.01 (4)
C14—C13—C12	119.4 (2)	O3^ii^—K—O3	180.00 (5)
C14—C13—H13	120.3	C36—O3—C35	111.99 (15)
C12—C13—H13	120.3	C36—O3—K	109.43 (12)
C13—C14—C15	120.76 (19)	C35—O3—K	109.30 (11)
C13—C14—H14	119.6	C38—O4—C37	113.48 (16)
C15—C14—H14	119.6	C38—O4—K	114.07 (11)
C14—C15—C16	119.9 (2)	C37—O4—K	114.16 (12)
C14—C15—H15	120.1	C34—O5—C39	112.95 (16)
C16—C15—H15	120.1	C34—O5—K	118.22 (11)
C11—C16—C15	119.81 (17)	C39—O5—K	117.03 (11)
C11—C16—N3	120.22 (16)	O5—C34—C35^ii^	108.25 (17)
C15—C16—N3	119.93 (18)	O5—C34—H34A	110.0
O1—C17—N3	122.09 (18)	C35^ii^—C34—H34A	110.0
O1—C17—C18	122.41 (18)	O5—C34—H34B	110.0
N3—C17—C18	115.49 (17)	C35^ii^—C34—H34B	110.0
C21—C18—C19	109.60 (19)	H34A—C34—H34B	108.4
C21—C18—C17	109.07 (18)	O3—C35—C34^ii^	110.08 (16)
C19—C18—C17	108.66 (18)	O3—C35—H35A	109.6
C21—C18—C20	110.6 (2)	C34^ii^—C35—H35A	109.6
C19—C18—C20	108.8 (2)	O3—C35—H35B	109.6
C17—C18—C20	110.01 (17)	C34^ii^—C35—H35B	109.6
C18—C19—H19A	109.5	H35A—C35—H35B	108.2
C18—C19—H19B	109.5	O3—C36—C37	110.25 (17)
H19A—C19—H19B	109.5	O3—C36—H36A	109.6
C18—C19—H19C	109.5	C37—C36—H36A	109.6
H19A—C19—H19C	109.5	O3—C36—H36B	109.6
H19B—C19—H19C	109.5	C37—C36—H36B	109.6
C18—C20—H20A	109.5	H36A—C36—H36B	108.1
C18—C20—H20B	109.5	O4—C37—C36	108.89 (18)
H20A—C20—H20B	109.5	O4—C37—H37A	109.9
C18—C20—H20C	109.5	C36—C37—H37A	109.9
H20A—C20—H20C	109.5	O4—C37—H37B	109.9
H20B—C20—H20C	109.5	C36—C37—H37B	109.9
C18—C21—H21A	109.5	H37A—C37—H37B	108.3
C18—C21—H21B	109.5	O4—C38—C39	109.51 (17)
H21A—C21—H21B	109.5	O4—C38—H38A	109.8
C18—C21—H21C	109.5	C39—C38—H38A	109.8
H21A—C21—H21C	109.5	O4—C38—H38B	109.8
H21B—C21—H21C	109.5	C39—C38—H38B	109.8
C23—C22—C27	118.63 (17)	H38A—C38—H38B	108.2
C23—C22—C6	120.80 (16)	O5—C39—C38	108.63 (17)
C27—C22—C6	120.55 (16)	O5—C39—H39A	110.0
C22—C23—C24	121.24 (19)	C38—C39—H39A	110.0
C22—C23—H23	119.4	O5—C39—H39B	110.0
C24—C23—H23	119.4	C38—C39—H39B	110.0
C25—C24—C23	119.5 (2)	H39A—C39—H39B	108.3
C25—C24—H24	120.3	H1O6—O6—H2O6	115 (3)
C23—C24—H24	120.3		

Symmetry codes: (i) −*x*+1, −*y*+2, −*z*+2; (ii) −*x*+1, −*y*+1, −*z*+1.

### Hydrogen-bond geometry (Å, º)

Cg1, Cg2, Cg3 and Cg5 are the centroids of the N1/C2–C5, N2/C7–C10, Co/N1/C2/C1/C10'/N2' and Co/N2/C10/C1'/C2'/N1' rings respectively.

**Table:**

*D*—H···*A*	*D*—H	H···*A*	*D*···*A*	*D*—H···*A*
N3—H*N*3···O6	0.88	2.09	2.966 (2)	172
O6—H1*O*6···N5^iii^	0.87	1.95 (2)	2.810 (3)	172 (2)
C20—H20*C*···O6	0.98	2.51	3.413 (3)	153
O6—H2*O*6···*Cg*2^i^	0.88	2.73 (2)	3.272 (2)	121 (2)
O6—H2*O*6···*Cg*3	0.88	2.81 (2)	3.455 (2)	131 (2)
O6—H2*O*6···*Cg*5^i^	0.88	2.81 (2)	3.455 (2)	131 (2)
C21—H21*B*···*Cg*1^iv^	0.98	2.82	3.737 (3)	156

Symmetry codes: (i) −*x*+1, −*y*+2, −*z*+2; (iii) −*x*+2, −*y*+2, −*z*+2; (iv) *x*+1, *y*, *z*.
